# Association of Glutamine and Glutamate Metabolism with Mortality among Patients at Nutritional Risk—A Secondary Analysis of the Randomized Clinical Trial EFFORT

**DOI:** 10.3390/nu16020222

**Published:** 2024-01-10

**Authors:** Carla Wunderle, Diana von Arx, Sydney Chiara Mueller, Luca Bernasconi, Peter Neyer, Pascal Tribolet, Zeno Stanga, Beat Mueller, Philipp Schuetz

**Affiliations:** 1 Division of General Internal and Emergency Medicine, University Department of Medicine, Kantonsspital Aarau, Tellstrasse 25, 5001 Aarau, Switzerland; carla.wunderle@ksa.ch (C.W.); beat.mueller@ksa.ch (B.M.); 2 Medical Faculty, University of Basel, 4056 Basel, Switzerland; 3 Faculty of Biomedical Scienes, Università della Svizzera italiana (USI), 6900 Lugano, Switzerland; 4 Institute of Laboratory Medicine, Kantonsspital Aarau, Tellstrasse 25, 5001 Aarau, Switzerlandpeter.neyer@ksa.ch (P.N.); 5 Department of Health Professions, Bern University of Applied Sciences, Murtenstrasse 10, 3008 Bern, Switzerland; 6 Faculty of Life Sciences, University of Vienna, Djerassiplatz 1, 1030 Vienna, Austria; 7 Department of Diabetes, Endocrinology, Nutritional Medicine and Metabolism (UDEM), Inselspital, Bern University Hospital, University of Bern, Freiburgstrasse 15, 3010 Bern, Switzerland

**Keywords:** malnutrition, polymorbid patient, individualized nutrition support, glutamine, glutamate, biomarker

## Abstract

Glutamine and its metabolite glutamate serve as the main energy substrates for immune cells, and their plasma levels drop during severe illness. Therefore, glutamine supplementation in the critical care setting has been advocated. However, little is known about glutamine metabolism in severely but not critically ill medical patients. We investigated the prognostic impact of glutamine metabolism in a secondary analysis of the *Effect of Early Nutritional Support on Frailty, Functional Outcomes, and Recovery of Malnourished Medical Inpatients Trial* (EFFORT), a randomized controlled trial comparing individualized nutritional support to usual care in patients at nutritional risk. Among 234 patients with available measurements, low plasma levels of glutamate were independently associated with 30-day mortality (adjusted HR 2.35 [95% CI 1.18–4.67, *p* = 0.015]). The impact on mortality remained consistent long-term for up to 5 years. No significant association was found for circulating glutamine levels and short- or long-term mortality. There was no association of glutamate nor glutamine with malnutrition parameters or with the effectiveness of nutritional support. This secondary analysis found glutamate to be independently prognostic among medical inpatients at nutritional risk but poorly associated with the effectiveness of nutritional support. In contrast to ICU studies, we found no association between glutamine and clinical outcome.

## 1. Introduction

Glutamine is a non-essential amino acid, which is vital for several key stress response pathways in critical illness [1,2]. Under certain conditions, glutamine becomes essential for the maintenance of metabolic functions in the context of glutathione synthesis, nitrogen exchange, and purine and pyrimidine productions and is therefore considered a conditionally essential amino acid. Additionally, glutamine serves as a main energy substrate for immune cells and cells of the gut-associated lymphatic tissue. Converted to glutamate and α-ketoglutarate, glutamine can boost energy production across diverse cell types via the Krebs cycle [3]. During a catabolic state or acute disease, some immune cells, such as lymphocytes and macrophages, consume even more glutamine than glucose [4,5]. In contrast, muscle cells, which are the main sites of glutamine synthesis, show reduced synthesis activity of the latter due to proteolysis, atrophy, and hypermetabolism. As a result, blood plasma glutamine levels are found to be depleted, which exacerbates the catabolic state [3]. 

Low plasma glutamine concentration is associated with increased mortality and morbidity [2,6,7,8]. Therefore, glutamine administration has been studied in various clinical settings, such as cancer and critical illness [9,10,11], or in patients suffering from burn injuries [12]. Glutamine supplementation was thereafter recommended for critically ill patients in the 2019 nutritional guidelines from the European Society for Clinical Nutrition and Metabolism [13]. However, a recent large, double-blind, randomized, placebo-controlled trial of 1209 burned patients showed no effect of glutamine supplementation on the time to discharge alive from the hospital [12]. Thus, there is still an ongoing controversy about the effectiveness of glutamine supplementation in the setting of critical illness.

Disease-related malnutrition (DRM) is a growing health concern among elderly and polymorbid medical inpatients, a condition that is strongly associated with increased mortality, complications, and reduced quality of life [14,15,16]. However, recent large trials [17,18] and a meta-analysis [19] have shown that nutritional support is an effective and cost-efficient [20] intervention to lower the risk of worse clinical outcomes [17,18,19,21,22]. Despite the overall beneficial effect of nutritional support, there are subgroups with certain clinical conditions, such as high inflammation [23], or severely ill patient populations [24] that show less benefit from nutritional treatment [23,24,25]. In fact, several studies have demonstrated that the acute-phase response and individual stress metabolism influence the way nutritional support affects the body [26,27]. In contrast to these findings, patients with impaired muscle strength [28] or advanced kidney failure [29] showed a more favorable response to nutritional support in medical inpatients [29]. A better understanding of the pathophysiology and DRM phenotype is key to improving nutritional strategies and will enable a more individualized approach. In addition to the current approach of selection for nutritional intervention using the nutritional history of patients, including changes in appetite and weight loss [19], other factors, such as severity of inflammation [23], specific comorbidities, and specific blood markers [30], might be helpful.

Despite thorough research on glutamine supplementation in the intensive care setting, little is known about glutamine metabolism in patients with severe but not critical illnesses, such as medical inpatients with acute disease. Herein, our aim was to investigate the roles of glutamine and its metabolite glutamate in predicting clinical outcomes regarding the response to nutritional support in a previous randomized controlled trial. 

## 2. Materials and Methods

### 2.1. Study Design and Participants

This was a secondary analysis of the EFFORT trial, which is a pragmatic, investigator-initiated, open-label, non-commercial, multicenter, randomized controlled trial that was undertaken in eight Swiss hospitals. The participating centers were either secondary or tertiary care hospitals, such as the University Clinic in Aarau, the University Hospital in Bern, the Cantonal hospitals in Lucerne, Solothurn, St. Gallen, Muensterlingen, and Baselland, and the regional hospital in Lachen. Hospitalized patients were screened using the Nutritional Risk Screening 2002 (NRS), which is a validated risk screening tool for malnutrition. This assesses the patient’s nutritional status (based on body mass index (BMI) and weight loss, as well as food intake) and disease severity. Patients received points from 0 to 3, depending on each risk predictor, and received an additional point if they were aged over 70 years. The inclusion criteria were defined as a minimum age of 18 years, an NRS total score of 3 points or greater, and an expected length of stay in the hospital for at least 5 days. Patients who were willing to give informed consent within 48 h of hospital admission were included. Patients were enrolled between 1 April 2014 and 28 February 2018. Exclusion criteria were an initial hospitalization in intensive care units or surgical wards; inability to ingest oral nutrition; already receiving nutritional support at admission; having a terminal condition; being admitted to hospital because of anorexia nervosa, acute pancreatitis, acute liver failure, cystic fibrosis, or stem cell transplantation; received gastric bypass surgery; received contraindications for nutritional support; and being previously included in the trial. The study protocol was approved by the Ethics Committee of Northwest and Central Switzerland (EKNZ) in January 2014 (registration ID 2014_001).

### 2.2. Randomization and Procedures

Patients were randomly assigned (1:1) to a control or intervention group. Nutritional support was initiated in the intervention group based on an individual nutritional treatment algorithm within 48 h after hospital admission. Control group patients received usual hospital food without further nutritional support or dietary counseling. They were allowed to eat according to their appetite. Participants and investigators were aware of group assignments at any time. However, structured telephone interviews to assess clinical outcomes were conducted by trained and blinded study nurses after discharge. Patients in the intervention group received individual nutrition therapy from trained, registered dietitians to reach protein and caloric targets. The daily protein target was set at 1.2–1.5 g/kg of bodyweight. A lower protein intake goal was defined for patients with acute renal failure (0.8 g/kg of bodyweight). The weight-adjusted Harris–Benedict equation was used to predict caloric requirements [31]. Trained, registered dietitians defined individualized goals for each patient. This plan was initially based on oral nutrition provided by the hospital kitchen (including food adjustment according to patient preferences, food fortification, such as enrichment of hospital food by adding protein powder, and snacks between meals) and oral nutritional supplements. A further increase in nutritional support to enteral or parenteral feeding was recommended if at least 75% of the daily caloric and protein targets could not be reached through oral feeding within 5 days. The nutritional algorithm used during the trial can be found in the original publication [17]. Nutritional intake was reassessed every 24–48 h throughout the hospital stay. Upon hospital discharge, the intervention was discontinued.

### 2.3. Analysis of Blood Biomarkers

Upon study inclusion, blood samples were collected, immediately processed, frozen in aliquots, and stored under a temperature controlled at −80 °C until further analysis. Admission plasma metabolites were analyzed from February to April 2019 by liquid chromatography coupled to tandem mass spectrometry (LC-MS/MS). An Ultimate 3000 UHPLC (Thermos Fisher, San Jose, CA, USA) system coupled to a Sciex QTRAP 5500 linear ion-trap quadrupole mass spectrometer (Sciex, Darmstadt, Germany) and the AbsoluteIDQ^®^ p180 kit (BIOCRATES Life Sciences AG, Innsbruck, Austria) were used [32,33,34]. An inter-laboratory assessment of this commercially available kit for targeted metabolomics showed the reliability of the metabolomics assay [35,36,37]. Measurements without a detectable signal for glutamine or glutamate were considered incorrect and were excluded in our analysis. Finally, we analyzed a total number of 234 glutamine and glutamate measurements. The glutamate to glutamine ratio was calculated as a surrogate to estimate the consumption of glutamine.

### 2.4. Outcomes

Our primary endpoint was defined as all-cause, short-term mortality measured at 30 days. The secondary endpoints were chosen as mid- and long-term mortality (at 180 days, 1 year, 2 years, 3 years, and 5 years); adverse events within 30 days; admission to the intensive care unit from the medical ward; non-elective hospital readmission after discharge; major complications, such as respiratory failure, a major cardiovascular event (i.e., stroke, intracranial bleeding, cardiac arrest, myocardial infarction, or pulmonary embolism), acute renal failure, and gastro-intestinal failure (i.e., hemorrhage, intestinal perforation, acute pancreatitis); decline in the functional status of more than 10% (measured by Barthel index); and total length of hospital stay and incidence of falls during the 180-day follow-up period. Additionally, nutritional outcomes were set as secondary endpoints, i.e., mean caloric and protein intakes per kg of bodyweight and achieving caloric and protein targets. Follow-up interviews for outcome assessment were performed at days 30 and 180 via phone calls. If necessary, family members or family physicians were contacted to verify the survival. 

### 2.5. Statistical Analysis

STATA 17.0 was used for statistical analysis. Statistical significance was tested at 95% confidence intervals (CI), corresponding to a *p*-value of 0.05. Continuous variables were expressed as mean ± standard deviation (SD), and binary and categorical variables were expressed as the number or count and percentages. The Liu method was used to calculate the empirical optimal cut-off values for the primary endpoint (30-day mortality) [38]. Patients in this secondary analysis were stratified into groups based on low or high glutamine or glutamate levels [39]. The cut-off concentration for glutamine was calculated to be 595.5 μmol/L, for glutamate, it was 167.5 μmol/L, and for their ratio, it was 0.28, which we then used to stratify them into high or low groups. A two-samples *t*-test was used to compare continuous variables, and Pearson’s chi-squared test was used for categorical and binary variables. Adjustment for potential confounding and random imbalances in regression analyses were made, including sex, baseline nutritional status (NRS total score), C-reactive protein (CRP), randomization group, and Carlson comorbidity index (CCI).

Associations between the metabolite levels and malnutrition parameters as well as secondary clinical outcomes were assessed using logistic regression models for the binary outcome, and for continuous outcomes, we used linear regression models, reported as the odds ratio (OR) and coefficient, respectively. Cox regression models were calculated for time-to-event analysis, with recorded hazard ratios (HR). The HR was calculated for all mortality endpoints (30 and 180 days and 1, 2, 3, and 5 years). Kaplan–Meier curves were used for the graphical display of the probability of all-cause mortality within 30 and 180 days.

Additionally, we investigated the response to nutritional therapy by comparing the hazards of mortality in the intervention vs. control groups, stratified by high versus low glutamine or glutamate plasma levels at admission. In all our analyses, the intention-to-treat principle was used. Limits to detect outliers were calculated by mean ±3 standard deviations of the sample (z-score method), and sensitivity analysis was performed for metabolites by comparing statistical results for data with and without outliers.

## 3. Results

### 3.1. Patient Population

**Figure 1:**
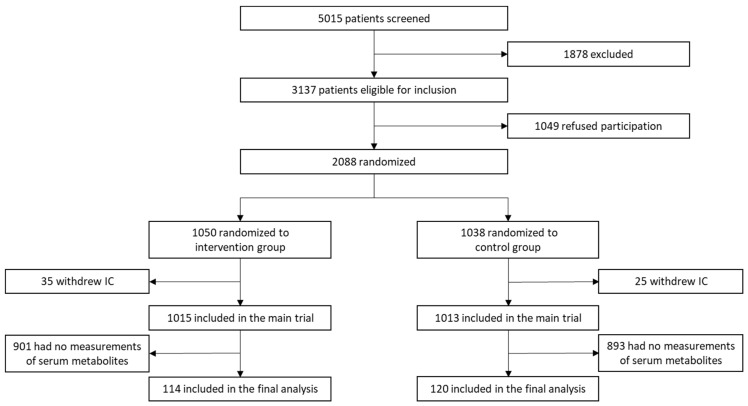
Study flow chart of the secondary analysis based on Schuetz et al., 2019 [17]; IC, informed consent.

We had full clinical and metabolomic data on 234 of 2028 patients (11.5%) that were included in our analyses (Figure 1). Of these, 159 patients had low plasma glutamine levels, and 160 patients had low plasma glutamate levels. Overall, the mean age of our cohort was 73.6 years (+/− 13.3 years), and 57.7% of patients were male. Overall, patients had a high burden of comorbidities, indicated by a CCI of 6.4. Most baseline characteristics, such as risk of malnutrition indicated by the NRS total score, were equally distributed. However, in the low-glutamine group, cancer as a main diagnosis was more prominent (58 [36.5%] vs. 16 [21.3%], *p* = 0.020), whereas diabetes as a comorbidity was less often observed (Table 1). On the other hand, stratified by high versus low glutamate, all baseline characteristics were equally distributed without any imbalances (Supplemental Table S1). 

### 3.2. Association of Glutamine and Glutamate with Nutritional Parameters and Inflammation

In the next step, we investigated the association of glutamine and glutamate levels and their ratio with different nutritional parameters, particularly the NRS total score, its components, the BMI, or CRP. Data for glutamine are presented below in Table 2. We did not find any significant results within the subgroups. Neither glutamine nor glutamate or their ratio showed any significant association with the NRS total score, its components, or baseline inflammation. The results remained consistent even in sensitivity analyses without outliners (Supplementary Tables S3 and S4). Data for glutamate can be found in the Supplementary Materials (Supplementary Table S2). 

### 3.3. Prognostic Value of Low Glutamine or Low Glutamate to Predict Clinical Outcomes

**Figure 2:**
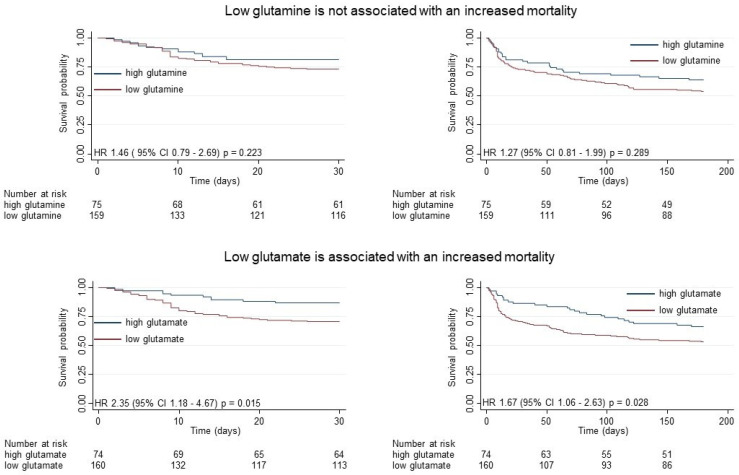
Kaplan–Meier estimate for 30-day and 180-day mortality according to high versus low glutamine (Gln) or glutamate (Glu) levels. Glutamine and glutamate levels at admission were stratified by the cut-off value (Gln 595.5 μmol/L; Glu 167.5 μmol/L). Low levels are defined as less than or equal to the cut-off value, and high levels are defined as greater than the cut-off value. All HR shown are adjusted for CCI, CRP, NRS total score, sex, and intervention.

Next, the associations of glutamine and glutamate with mortality and other predefined clinical outcomes were assessed (Table 3). We found no significant differences in short- or long-term mortality in patients with low versus high glutamine plasma concentrations. However, patients with low glutamate plasma concentrations had more than a doubling in 30-day mortality, resulting in an unadjusted HR of 2.40 (95% CI = 1.21–4.75, *p* = 0.012). These results remained robust in an adjusted model for CCI, CRP, the NRS total score, sex, and the randomization group, with an HR of 2.35 (95% CI = 1.18–4.67, *p =* 0.015). Additionally, the results stayed consistent for long-term mortality at 180 days up to 5 years (adjusted HR: 1.67, 95% CI: 1.06–2.63, and *p* = 0.028, respectively). Figure 2 visualizes the survival probability in Kaplan–Meier curves for 30- and 180-day all-cause mortality among the population with high and low glutamine or glutamate.

For secondary clinical outcomes, we observed similar trends to mortality. While there was only a little difference between high- and low-glutamine groups, we found an association between low glutamate levels and the composite endpoint of adverse clinical outcomes within 30 days (adjusted HR: 2.65, 95% CI: 1.39–5.05, *p* = 0.003), as well as major complications (adjusted HR: 5.35, 95% CI: 1.21–23.67, *p* = 0.027). Full results can be found in Supplemental Tables S5–S9.

### 3.4. Association of Glutamine and Glutamate Levels or the Ratio with the Effectiveness of Nutritional Support

**Figure 3:**
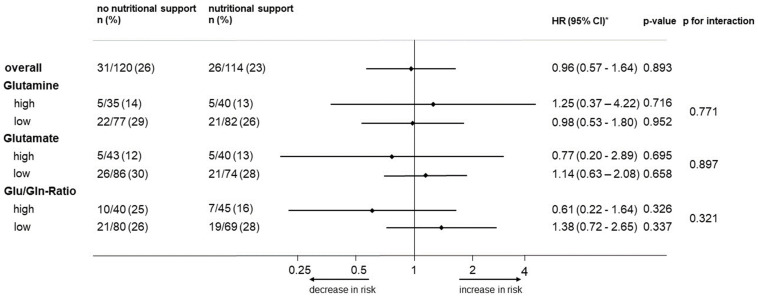
Forest plot for 30-day mortality and subgroup analysis for response to nutritional support. HR, hazard ratio. * Adjusted for CCI, CRP, NRS total score, sex, and intervention.

To assess whether the response to nutritional support would differ according to baseline glutamine or glutamate levels or their ratio, we compared the effects of nutritional support on 30-day all-cause mortality among patients randomized to intervention versus the control group with high and low glutamine and glutamate or their ratio, respectively (Figure 3). The 30-day mortality did not differ in subgroups of patients according to their plasma glutamine and glutamate levels, resulting in a non-significant interaction analysis (*p* = 0.771 for high versus low glutamine, *p* = 0.897 for high versus low glutamate, and *p* = 0.321 for glutamate/glutamine ratio). 

## 4. Discussion

This secondary analysis of a randomized trial investigating possible implications of glutamine, glutamate, and their ratio, respectively, regarding outcome and treatment response has several key findings. First, among medical inpatients at nutritional risk, neither glutamine nor glutamate are well correlated with established nutritional markers and thus may not be viewed as markers for malnutrition. Second, patients with low glutamine levels did not show an increased mortality, whereas patients with low glutamate levels had a significantly higher risk of dying within 30 days. Glutamate was also a strong prognostic marker for other adverse outcomes. Our findings remained robust in different statistical models adjusted for possible confounders. Third, low glutamine levels, low glutamate levels, or a low ratio of glutamate to glutamine were not associated with a more pronounced response to individualized nutritional therapy in terms of 30-day mortality. Therefore, these metabolites may not be considered predictive markers of response to nutritional treatment. Several of these findings need further comment.

We found no association between low plasma levels of glutamine or glutamate and several malnutrition parameters. Nevertheless, glutamine was low in our cohort, compared to a healthy French cohort [35] that was using the same analysis kit (mean glutamine plasma level 522.5 µmol/L versus 627.9 µmol/L) and to a cancer patient cohort (522.5 µmol/L versus 574.0 µmol/L) [9]. This finding is in line with previous trials that showed glutamine depletion during severe illness and under catabolic conditions [2,6,7,40]. However, low glutamine levels were not associated with a worse clinical outcome, which differs from previous data from the ICU or after major surgery [7]. This may be explained by the less severely ill patient cohort, in which glutamine depletion may also have been less severe and, thus, had no negative implications for the clinical outcome. While most studies in the ICU show that low glutamine levels are associated with poorer clinical outcomes, there is also evidence that high glutamine levels are associated with increased mortality, mainly due to impaired hepatic glucose metabolism [41]. We could not find this U shape in our data, and we assumed that most patients had a functioning glucose metabolism because we did not include critically ill patients and had liver failure as an exclusion criterion. Regarding glutamate levels, we observed increased concentrations in our patient population: the average glutamate level was 152.1 µmol/L. This is about three times higher, as compared to 46.2 µmol/L in healthy French subjects and 63.2 µmol/L in cancer patients, respectively. Since our population was polymorbid and at nutritional risk, we assumed that high plasma glutamate levels could result from high degradation and consumption rates of glutamine, a phenomenon that was observed in secondary analyses investigating cardiometabolic risk and diabetes [42] but not in malnourished patients from the large NOURISH trial [43], where glutamate was also low. However, we found that patients with high glutamate levels had a survival benefit, a finding contrary to findings from ICU trials [44] but in line with a secondary analysis of medical inpatients [43]. Metabolic pathways are complex and can be influenced by several clinical conditions. Since we found high glutamate levels, we assumed that the deamination of glutamate to α-ketoglutarate by the glutamate dehydrogenase and feeding into the Krebs cycle was the limiting step in energy production by glutamine, which is the upstream metabolite.

We found substantially more patients with low glutamine levels (159 low glutamine vs. 75 high glutamine) in our patient population. This is consistent with evidence that glutamine is depleted in conditions of high inflammation, cancer [9], and infection [4], as well as in patients with burn injuries, which is associated with weaker immune system performance [45]. Therefore, several trials have investigated whether glutamine supplementation, as recommended in various guidelines [13,46], has a benefit on clinical outcomes, such as mortality and rehospitalization, due to its immune-enhancing effects [45]. However, the most recent multicenter, double-blind, randomized control trial by Heyland et al. in 2022 failed to show the benefit of enteral guideline-compliant glutamine supplementation in severe burn patients when accounting for time to discharge alive from the hospital, 6-month mortality, or the occurrence of bacteremia [12]. Besides this study, there were some smaller trials [11] demonstrating the beneficial effect of glutamine supplementation. Nevertheless, a systematic review and meta-analysis confirmed that supplementation has no effect on the reduction in hospital mortality, infectious complications, or the intensive care unit stay in patients with critical illness [10]. Whether glutamine supplementation in less severely ill and malnourished medical inpatients would be beneficial remains unclear and needs further investigations. Although we found elevated glutamate levels in our patient population, high glutamate levels were also associated with better survival. Therefore, the role of glutamate supplementation remains unclear.

We also investigated the response to nutritional interventions as a function of glutamine or glutamate levels, yet no difference in the efficacy of nutritional support was found between patients with high and low glutamine or glutamate concentrations. Our findings, thus, do not support the measurement of these metabolites to explain variability in response to treatment and to further personalize nutritional support.

### Strengths and Limitations

The greatest strengths of this study include the well-characterized patient cohort, the randomized design, and the prospectively collected short- and long-term outcomes. This allowed us to adjust our analysis for potential confounders, such as comorbidities and nutritional parameters, and we obtained consistent results. Still, as this study is a secondary analysis, the results should be viewed as exploratory and hypothesis-generating, rather than definite, requiring confirmation in larger prospective samples. We are aware of additional limitations, including the design of our subgroup analysis at a single center, which results in lower statistical power and lower external validity. Additionally, we measured glutamine and glutamate plasma levels at admission only, so the dynamics over time and the effect of nutritional support on metabolite remains unclear. There are also restraints regarding the metabolomic kit used. So far, it has mainly been used for research purposes, and there is a lack of well-validated reference values.

## 5. Conclusions

This secondary analysis of a prospective randomized trial found glutamate to be an independent prognostic parameter among medical inpatients at nutritional risk, but it was poorly associated with the effectiveness of nutritional support. In contrast to findings from intensive care, low glutamine levels in medical inpatients were not associated with worse clinical outcomes. A better understanding of glutamine metabolism may help to further improve risk assessment for unfavorable outcomes in medical patients at nutritional risk. In addition, the effect of glutamine or glutamate supplementation in this less severely ill patient population remains an open question for further research.

## Figures and Tables

**Table 1:** Baseline characteristics overall and stratified by high versus low glutamine.

	Overall (n = 234)	Low Glutamine (n = 159)	High Glutamine (n = 75)	*p*-Value
Sociodemographic				
Male sex	135 (57.7%)	87 (54.7%)	48 (64.0%)	0.18
Mean age in years (SD)	73.6 (13.3)	73.5 (13.6)	73.9 (13)	0.81
Nutritional assessment				
Mean body mass index in kg/m^2^ (SD)	24 (5)	24 (5)	25 (5)	0.32
Mean bodyweight in kg (SD)	69 (15)	68 (15)	71 (14)	0.11
Mean height in cm (SD)	168.1 (8.6)	167.5 (8.6)	169.3 (8.4)	0.15
NRS total score				0.75
3 points	60 (25.6%)	39 (24.5%)	21 (28.0%)	
4 points	79 (33.8%)	56 (35.2%)	23 (30.7%)	
≥5 points	95 (40.6%)	64 (40.3%)	31 (41.3%)	
CRP, day 1, mg/L	84.1 (79.8)	85.1 (78.2)	82.0 (83.4)	0.78
Admission diagnosis				
Infection	63 (26.9%)	40 (25.2%)	23 (30.7%)	0.38
Cancer	74 (31.6%)	58 (36.5%)	16 (21.3%)	**0.020**
Cardiovascular disease	24 (10.3%)	15 (9.4%)	9 (12.0%)	0.55
Frailty	13 (5.6%)	11 (6.9%)	2 (2.7%)	0.19
Lung disease	10 (4.3%)	6 (3.8%)	4 (5.3%)	0.58
Gastrointestinal disease	13 (5.6%)	9 (5.7%)	4 (5.3%)	0.92
Renal disease	15 (6.4%)	9 (5.7%)	6 (8.0%)	0.50
Comorbidity				
Charlson Comorbidity Index	6.4 (2.8%)	6.4 (2.9%)	6.3 (2.7%)	0.76
Hypertension	137 (58.5%)	92 (57.9%)	45 (60.0%)	0.76
Malignant disease	110 (47.0%)	77 (48.4%)	33 (44.0%)	0.53
Chronic kidney disease	81 (34.6%)	51 (32.1%)	30 (40.0%)	0.23
Coronary heart disease	54 (23.1%)	36 (22.6%)	18 (24.0%)	0.82
Diabetes mellitus	43 (18.4%)	23 (14.5%)	20 (26.7%)	**0.025**
Congestive heart failure	45 (19.2%)	33 (20.8%)	12 (16.0%)	0.39
Chronic obstructive pulmonary disease	26 (11.1%)	20 (12.6%)	6 (8.0%)	0.30
Peripheral arterial disease	26 (11.1%)	16 (10.1%)	10 (13.3%)	0.46
Cerebrovascular disease	27 (11.5%)	17 (10.7%)	10 (13.3%)	0.56
Dementia	11 (4.7%)	8 (5.0%)	3 (4.0%)	0.73
Metabolites				
Mean plasma glutamine concentration (μmol/L)	522.5 (196.2)	423.4 (130.2)	732.7 (138.3)	<0.001
Mean plasma glutamate concentration (μmol/L)	152.10 (110.35)	166.34 (124.38)	121.91 (62.87)	0.004

SD, standard derivation; NRS, nutritional risk screening 2002; CRP, C-reactive protein.

**Table 2:** Association of nutritional parameters with a decrease in plasma glutamine levels.

	Unadjusted	Adjusted *
Glutamine (decrease of 10 μmol/L)	Coef (95% CI) *p*-value	Coef (95% CI) *p*-value
Nutritional assessment		
Bodyweight (kg)	0.06 (−0.11 to 0.23) *p =* 0.459	0.06 (−0.11 to 0.24) *p =* 0.474
Body mass index (kg/m^2^)	0.13 (−0.38 to 0.64) *p =* 0.620	0.12 (−0.39 to 0.63) *p =* 0.640
NRS total score		
3 points	reference	reference
4 points	−4.56 (−11.15 to 2.04) *p =* 0.175	−4.80 (−11.51 to 1.91) *p =* 0.160
≥5 points	−1.45 (−7.77 to 4.87) *p =* 0.651	−2.47 (−8.96 to 4.01) *p =* 0.453
CRP, day 1, mg/L	0.00 (−0.03 to 0.03) *p =* 0.948	−0.01 (−0.08 to 0.05) *p =* 0.659
NRS score components		
Loss of appetite **	−3,99 (−11.66 to 3.67) *p =* 0.306	−5.82 (−13.80 to 2.15) *p =* 0.152
Bodyweight loss (kg)		
<5% in 3 months	reference	reference
>5% in 3 months	6.00 (−1.03 to 13.03) *p =* 0.094	6.07 (−0.99 to 13.14) *p =* 0.092
>5% in 2 months	−3.83 (0.293 to −11.00) *p =* 0.293	−4.25 (−11.44 to 2.93) *p =* 0.245
>5% in 1 month	−3.94 (−10.52 to 2.64) *p =* 0.239	−3.55 (−10.18 to 3.07) *p =* 0.292
Reduced dietary intake **		
>75%	reference	reference
50–75%	−5.54 (−14.72 to 3.65) *p =* 0.236	−5.57 (−14.83 to 3.69) *p =* 0.237
25–50%	−7.27 (−16.00 to 1.47) *p =* 0.102	−6.95 (−15.83 to 1.92) *p =* 0.124
<25%	−0.14 (−9.77 to 9.49) *p =* 0.977	−0.26 (−9.98 to 9.47) *p =* 0.958
Severity of disease		
1	reference	reference
2	−2.89 (−8.16 to 2.38) *p =* 0.281	−3.29 (−8.82 to 2.25) *p =* 0.243
3	−7.04 (−45.16 to 31.09) *p =* 0.716	−7.69 (−46.53 to 31.14) *p =* 0.697

Unadjusted and adjusted regression analyses were performed to identify associations of glutamine concentrations at admission with nutritional parameters. The regression coefficients (95% CI) indicate the change in glutamine concentration by ten units (10 μmol/L). For binary parameters, patients with the characteristics are compared to patients without the characteristic. BMI, body mass index; NRS 2002, Nutritional Risk Screening 2002; CRP, C-reactive protein; CI, confidence interval; Coef, coefficient. * Adjusted for CCI, CRP, sex, and intervention. ** In the week preceding hospitalization compared to usual appetite and intake.

**Table 3:** Prognostic values of high vs. low levels of glutamine or glutamate on mortality.

			Unadjusted	Adjusted *
Short- and Long-Term Mortality	n. of Event (%)	n. of Event (%)	HR (95% CI) *p*-Value	HR (95% CI) *p*-Value
30-day all-cause mortality	high	low		
Glutamine	14/75 (19%)	43/159 (27%)	1.50 (0.82 to 2.74) *p =* 0.189	1.46 (0.79 to 2.69) *p =* 0.223
Glutamate	10/74 (14%)	47/160 (29%)	2.40 (1.21 to 4.75) *p =* 0.012	2.35 (1.18 to 4.67) *p =* 0.015
180-day all-cause mortality				
Glutamine	25/75 (36%)	73/159 (46%)	1.37 (0.88 to 2.13) *p =* 0.163	1.27 (0.81 to 1.99) *p =* 0.289
Glutamate	25/74 (34%)	75/160 (47%)	1.64 (1.05 to 2.59) *p =* 0.031	1.67 (1.06 to 2.63) *p =* 0.028
1-year all-cause mortality				
Glutamine	31/75 (41%)	83/159 (52%)	1.39 (0.92 to 2.10) *p =* 0.119	1.31 (0.86 to 1.99) *p =* 0.203
Glutamate	28/74(38%)	86/160 (54%)	1.68 (1.10 to 2.58) *p =* 0.017	1.69 (1.10 to 2.59) *p =* 0.017
2-year all-cause mortality				
Glutamine	41/75 (55%)	95/159 (60%)	1.23 (0.85 to 1.77) *p =* 0.269	1.20 (0.83 to 1.74) *p =* 0.331
Glutamate	33/74 (45%)	103/160 (64%)	1.76 (1.19 to 2.60) *p =* 0.005	1.78 (1.20 to 2.64) *p =* 0.004
3-year all-cause mortality				
Glutamine	41/75 (55%)	99/159 (62%)	1.28 (0.89 to 1.84) *p =* 0.191	1.25 (0.86 to 1.80) *p =* 0.238
Glutamate	38/74 (51%)	102/160 (64%)	1.53 (1.05 to 2.22) *p =* 0.025	1.57 (1.08 to 2.28) *p =* 0.019
5-year all-cause mortality				
Glutamine	45/75 (60%)	108/159 (68%)	1.28 (0.90 to 1.81) *p =* 0.167	1.24 (0.87 to 1.77) *p =* 0.224
Glutamate	46/74 (62%)	107/160 (67%)	1.38 (0.98 to 1.96) *p =* 0.067	1.42 (1.00 to 2.01) *p =* 0.050

Cox regression models reporting adjusted hazard ratios according to levels stratified by cut-off values of glutamine (595.5 μmol/L) and glutamate (167.5 μmol/L). Low levels are defined as less than or equal to the cut-off value, and high levels are defined as greater than the cut-off value. Abbreviations: HR, hazard ratio; CI, confidence interval. * Adjusted for CCI, CRP, NRS total score, sex, and intervention.

## Data Availability

The data presented in this study are available on request from the corresponding author. The data are not publicly available due to ongoing investigations.

## Supplementary Materials

The following supporting information can be downloaded at: https://www.mdpi.com/article/10.3390/nu16020222/s1, Table S1: Baseline characteristics overall and stratified by high and low glutamate. Table S2: Association of nutritional parameters with a decrease in plasma glutamate levels. Table S3: Association of nutritional parameters with a decrease in plasma glutamate levels (without outliers). Table S4: Association of nutritional parameters with a decrease in plasma glutamate levels (logarithmic calculation). Table S5: Prognostic value of high vs. low levels of glutamine or glutamate on secondary clinical outcomes. Table S6: Prognostic value of high vs. low levels of glutamine or glutamate on secondary clinical outcomes. Table S7: Prognostic value of high vs. low glutamate-to-glutamine-ratio on mortality. Table S8: Prognostic value of high vs. low glutamate-to-glutamine-ratio on secondary clinical outcomes. Table S9: Prognostic value of high vs. low glutamate-to-glutamine-ratio on secondary clinical outcomes.

## Abbreviations

ATP	adenosine triphosphate
BMI	body mass index
CCI	Charlson comorbidity index
CI	confidence interval
CRP	C-reactive protein
DRM	disease-related malnutrition
EFFORT	Effect of Early Nutritional Support on Frailty, Functional Outcomes, and Recovery of Malnourished Medical Inpatients Trial
EKNZ	Ethics Committee of Northwest and Central Switzerland
GA	glutaminase
HR	hazard ratio
LC-MS/MS	liquid chromatography coupled to tandem mass spectrometry
NH3	ammonia
NRS	nutritional risk screening 2002
OR	odds ratio
SD	standard deviation
UHPLC	ultra-high-pressure liquid chromatography
