# Supplementary material for: Association of Glutamine and Glutamate Metabolism with Mortality among Patients at Nutritional Risk—A Secondary Analysis of the Randomized Clinical Trial EFFORT
Source: Nutrients. 2024 Jan 10;16(2):222. doi: 10.3390/nu16020222 (PMC10821126; doi:10.3390/nu16020222)
Supplement: Supplementary file 1 [file nutrients-16-00222-s001.zip › nutrients-2787685-supplementary.pdf]

Supplemental Table S1: Baseline characteristics overall and stratified by high and low glutamate

	**overall (n=234)**	**low glutamate (n=160)**	**high glutamate (n=74)**	**p-value**
**Sociodemographic**				
Male sex	135 (57.7%)	94 (58.8%)	41 (55.4%)	0.63
Mean age in years (SD)	73.6 (13.3)	73.2 (12.6)	74.5 (14.9)	0.50
**Nutritional assessment**				
Mean body-mass-index in kg/m^2 (SD)	24 (5)	24 (5)	25 (5)	0.44
Mean bodyweight in kg (SD)	69 (15)	68 (15)	70 (15)	0.35
Mean height in cm (SD)	168.1 (8.6)	167.9 (8.0)	168.5 (9.7)	0.65
**NRS 2002 total score**				0.46
3 points	60 (25.6%)	38 (23.8%)	22 (29.7%)	
4 points	79 (33.8%)	53 (33.1%)	26 (35.1%)	
≥ 5 points	95 (40.6%)	69 (43.1%)	26 (35.1%)	
CRP, day 1, mg/l	84.1 (79.8)	86.9 (82.9)	78.1 (72.6)	0.44
**Admission diagnosis n (%)**				
Infectious disease	63 (26.9%)	46 (28.7%)	17 (23.0%)	0.35
Cancer disease	74 (31.6%)	52 (32.5%)	22 (29.7%)	0.67
Cardiovascular disease	24 (10.3%)	15 (9.4%)	9 (12.2%)	0.51
Frailty	13 (5.6%)	8 (5.0%)	5 (6.8%)	0.59
Lung disease	10 (4.3%)	7 (4.4%)	3 (4.1%)	0.91
Gastrointestinal disease	13 (5.6%)	8 (5.0%)	5 (6.8%)	0.59
Renal disease	15 (6.4%)	10 (6.2%)	5 (6.8%)	0.88
**Comorbidities n (%)**				
Charlson Comorbidity Index	6.4 (2.8)	6.4 (2.9)	6.2 (2.6)	0.69
Hypertension	137 (58.5%)	89 (55.6%)	48 (64.9%)	0.18
Malignant disease	110 (47.0%)	80 (50.0%)	30 (40.5%)	0.18
Chronic kidney disease	81 (34.6%)	52 (32.5%)	29 (39.2%)	0.32
Coronary heart disease	54 (23.1%)	33 (20.6%)	21 (28.4%)	0.19
Diabetes mellitus	43 (18.4%)	29 (18.1%)	14 (18.9%)	0.88
Congestive heart failure	45 (19.2%)	28 (17.5%)	17 (23.0%)	0.32
Chronic obstructive pulmonary disease	26 (11.1%)	16 (10.0%)	10 (13.5%)	0.43
Peripheral arterial disease	26 (11.1%)	15 (9.4%)	11 (14.9%)	0.21
Stroke	27 (11.5%)	18 (11.2%)	9 (12.2%)	0.84
Dementia	11 (4.7%)	7 (4.4%)	4 (5.4%)	0.73
**Metabolites**				
Mean plasma glutamine concentration (µmol/l)	522.5 (196.2)	567.5 (180.1)	425.2 (195.5)	<0.001
Mean plasma glutamate concentration (µmol/l)	152.10 (110.35)	97.18 (32.72)	270.85 (125.02)	<0.001

Table S2: Association of nutritional parameters with a decrease in plasma glutamate levels

	**unadjusted**	**adjusted***
**Glutamate decrease of 10 µmol/l**	Coef (95% CI) p-value	Coef (95% CI) p-value
**Nutritional assessment**		
Bodyweight in kg	-0.01 (-0.10 to 0.09) p= 0.879	0.00 (-0.10 to 0.10) p= 0.928
Body-mass-index in kg/m^2	-0.03 (-0.32 to 0.26) p= 0.848	-0.03 (-0.32 to 0.26) p= 0.846
**NRS total score**		
3 points	reference	reference
4 points	-0.53 (-4.23 to 3.16) p= 0.777	-0.49 (-4.29 to 3.31) p= 0.800
≥ 5 points	-3.75 (-7.30 to -0.19) p= 0.039	-3.78 (-7.46 to -0.10) p= 0.044
CRP, day 1, mg/l	0.00 (-0.02 to 0.02) p= 0.820	0.00 (-0.04 to 0.03) p= 0.802
**NRS score components**		
**Loss of appetite ****	-1.24 (-5.56 to 3.08) p= 0.572	-1.27 (-5.73 to 3.20) p= 0.577
**Weight loss**		
< 5% in 3 months	reference	reference
> 5% in 3 months	-0.80 (-4.84 to 3.23) p= 0.695	-0.74 (-4.84 to 3.36) p= 0.722
> 5% in 2 months	2.03 (-2.05 to 6.10) p= 0.328	2.03 (-2.09 to 6.15) p= 0.333
> 5% in 1 month	0.41 (-3.41 to 4.22) p= 0.834	0.37 (-3.51 to 4.24) p= 0.853
**Reduced dietary intake**		
>75%	reference	reference
50-75%	3.69 (-1.57 to 8.94) p= 0.168	3.78 (-1.55 to 9.12) p= 0.164
25-50%	1.02 (-4.00 to 6.05) p= 0.688	1.10 (-4.04 to 6.24) p=0.673
<25%	0.05 (-5.49 to 5.59) p= 0.987	0.14 (-5.50 to 5.78) p= 0.961
**Severity of disease**		
1	reference	reference
2	-1.42 (-4.41 to 1.57) p= 0.351	-1.52 (-4.69 to 1.66) p= 0.348
3	-2.62 (-24.48 to 19.25) p= 0.814	-3.60 (-26.08 to 18.87) p= 0.752

*Unadjusted and adjusted regression analysis to identify associations of glutamate concentration at admission with nutritional parameters. The regression coefficients (95% CI) indicate the change in glutamine concentration by ten units (10 µmol/l). For binary parameters, patients with the characteristics are compared to patients without the characteristic. BMI, body mass index; CI, Confidence Interval; Coef, Coefficient; NRS, Nutritional Risk Screening 2002; CRP, C-reactive protein; CI, confidence interval. *Adjusted for CCI, CRP, sex and intervention. ** In the week preceding hospitalisation compared to usual appetite and intake.*

Table S3: Association of nutritional parameters with a decrease in plasma glutamate levels (without outliers)

	**unadjusted**	**adjusted***
**Glutamate decrease of 10 µmol/l**	Coef (95% CI) p-value	Coef (95% CI) p-value
**Nutritional assessment**		
Bodyweight in kg	0.02 (-0.06 to 0.09) p= 0.612	0.02 (-0.06 to 0.10) p= 0.677
Body-mass-index in kg/m2	0.09 (-0.14 to 0.32) p= 0.430	0.08 (-0.15 to 0.31) p= 0.478
**NRS 2002 score**		
3 points	reference	reference
4 points	-0.04 (-2.96 to 2.88) p= 0.978	0.02 (-2.98 to 3.01) p= 0.992
≥ 5 points	-1.94 (-4.74 to 0.86) p= 0.173	-1.73 (-4.62 to 1.17) p= 0.241
CRP, day 1, mg/l	0.00 (-0.01 to 0.01) p= 0.917	0.00 (-0.03 to 0.03) p= 0.969
**NRS score components**		
**Loss of appetite ****	2.34 (-1.10 to 5.78) p= 0.182	2.55 (-1.01 to 6.11) p= 0.160
**Weight loss**		
< 5% in 3 months	reference	reference
> 5% in 3 months	-1.31 (-4.47 to 1.85) p= 0.415	-1.29 (-4.49 to 1.90) p= 0.426
> 5% in 2 months	-0.35 (-3.57 to 2.87) p= 0.830	-0.30 (-3.55 to 2.94) p= 0.854
> 5% in 1 month	0.96 (-1.99 to 3.92) p= 0.522	0.85 (-2.15 to 3.85) p= 0.577
**Reduced dietary intake**		
>75%	reference	reference
50-75%	1.44 (-2.68 to 5.56) p= 0.491	1.58 (-2.59 to 5.75) p= 0.457
25-50%	0.38 (-3.54 to 4.29) p= 0.850	0.44 (-3.55 to 4.44) p= 0.828
<25%	0.05 (-4.27 to 4.36) p= 0.983	0.17 (-4.21 to 4.55) p= 0.940
**Severity of disease**		
1	reference	reference
2	-2.01 (-4.34 to 0.31) p= 0.090	-2.37 (-4.82 to 0.09) p= 0.059
3	-1.90 (-18.75 to 14.95) p= 0.824	-3.88 (-21.13 to 13.36) p= 0.658

*Unadjusted and adjusted regression analysis to identify associations of glutamate concentration at admission with nutritional parameters. The regression coefficients (95% CI) indicate the change in glutamine concentration by ten units (10 µmol/l). For binary parameters, patients with the characteristics are compared to patients without the characteristic. BMI, body mass index; NRS 2002, Nutritional Risk Screening 2002; CRP, C-reactive protein; CI, confidence interval. *Adjusted for CCI, CRP, sex and intervention. ** In the week preceding hospitalisation compared to usual appetite and intake.*

Supplemental Table S4: Association of nutritional parameters with a decrease in plasma glutamate levels (logarithmic calculation)

	**unadjusted**	**adjusted***
**Glutamate decrease of 10 µmol/l**	Coef (95% CI) p-value	Coef (95% CI) p-value
**nutritional assessment**		
bodyweight in kg	0 (0.00 to 0.01) p= 0.750	0.00 (0.00 to 0.01) p= 0.761
body-mass-index in kg/m^2	0.00 (-0.01 to 0.02) p= 0.717	0.00 (-0.01 to 0.02) p= 0.745
**NRS 2002 score**		
3 points	reference	reference
4 points	-0.05 (-0.25 to 0.16) p= 0.653	-0.05 (0.00 to 0.00) p= 0.955
≥ 5 points	-0.21 (-0.40 to -0.01) p= 0.035	-0.20 (-0.40 to -0.00) p= 0.047
CRP, day 1, mg/l	0.00 (0.00 to 0.00) p= 0.872	0.00 (0.00 to 0.00) p= 0.955
**NRS score components**		
**loss of appetite** **	-0.02 (-0.25 to 0.22) p= 0.900	-0.01 (-0.26 to 0.23) p= 0.921
**bodyweight loss (kg)**		
< 5% in 3 months	reference	reference
> 5% in 3 months	-0.05 (-0.27 to 0.17) p= 0.659	-0.05 (-0.27 to 0.17) p= 0.661
> 5% in 2 months	0.03 (-0.19 to 0.26) p= 0.774	0.04 (-0.19 to 0.26) p= 0.753
> 5% in 1 month	0.01 (-0.20 to 0.22) p= 0.905	0.00 (-0.21 to 0.22) p= 0.971
**reduced dietary intake** **		
>75%	reference	reference
50-75%	0.13 (-0.16 to 0.42) p= 0.370	0.14 (-0.15 to 0.43) p= 0.355
25-50%	0.00 (-0.27 to 0.28) p= 0.976	0.00 (-0.28 to 0.29) p= 0.974
<25%	-0.14 (-0.32 to 0.28) p= 0.899	-0.14 (-0.32 to 0.29) p= 0.928
**severity of disease**		
1	reference	reference
2	-0.13 (-0.29 to 0.03) p= 0.123	-0.14 (-0.31 to 0.03) p= 0.111
3	0.00 (-1.19 to 1.18) p= 0.996	-0.07 (-1.29 to 1.15) p= 0.915

*Unadjusted and adjusted regression analysis to identify associations of glutamate concentration at admission with nutritional parameters. The regression coefficients (95% CI) indicate the change in glutamine concentration by ten units (10 µmol/l). For binary parameters, patients with the characteristics are compared to patients without the characteristic. BMI, body mass index; NRS 2002, Nutritional Risk Screening 2002; CRP, C-reactive protein; CI, confidence interval. *Adjusted for CCI, CRP, sex and intervention. ** In the week preceding hospitalisation compared to usual appetite and intake.*

Supplemental Table S5: Prognostic value of high vs. low levels of glutamine or glutamate on secondary clinical outcomes

	n. of event (%)	n. of event (%)	unadjusted	adjusted*
**Secondary clinical outcomes**	**high**	**low**	HR (95% CI) p-value	HR (95% CI) p-value
**Adverse events, 30 days**				
Glutamine	29/75 (39%)	62/159 (39%)	1.01 (0.58 to 1.78) p= 0.962	1.02 (0.56 to 1.85) p= 0.951
Glutamate	18/74 (24%)	73/160(46%)	2.16 (1.41 to 4.83) p= 0.002	2.65 (1.39 to 5.05) p= 0.003
**ICU, 30 days**				
Glutamine	1/75 (1%)	3/159 (2%)	1.42 (0.15 to 13.91) p= 0.762	1.51 (0.15 to 15.48) p= 0.729
Glutamate	0/74 (0%)	4/160 (3%)	1.00	1.00
**Rehospitalisation 30 days**				
Glutamine	8/75 (11%)	10/159 (6%)	0.56 (0.21 to 1.49) p= 0.246	0.57 (0.21 to 1.52) p= 0.263
Glutamate	3/74 (4%)	15/160 (9%)	2.45 (0.69 to 8.73) p= 0.168	2.41 (0.67 to 8.66) p= 0.177
**Major complications**				
Glutamine	8/75 (11%)	14/159 (9%)	0.81 (0.32 to 2.02) p= 0.649	0.80 (0.32 to 2.03) p= 0.641
Glutamate	2/74 (3%)	20/160 (13%)	5.14 (1.17 to 22.62) p= 0.030	5.35 (1.21 to 23.67) p= 0.027
**Decline in functional status of >10%**				
Glutamine	19/75 (25%)	45/159 (28%)	1.16 (0.62 to 2.17) p= 0.635	1.17 (0.60 to 2.30) p= 0.643
Glutamate	14/75 (19%)	50/160 (31%)	1.95 (1.00 to 3.81) p= 0.051	1.95 (0.95 to 4.02) p= 0.069
**Falls, 180 days**				
Glutamine	5/75 (7%)	18/158 (11%)	1.80 (0.64 to 5.05) p= 0.264	1.69 (0.59 to 4.82) p= 0.327
Glutamate	6/74 (8%)	17/159 (11%)	1.36 (0.51 to 3.60) p= 0.539	1.47 (0.54 to 3.99) p= 0.452

*Cox regression models reporting adjusted hazard ratios according to levels stratified by cutoff values of glutamine (595.5 µmol/l) and glutamate (167.5 µmol/l). Low levels are defined as less or equal the cutoff value, high levels as greater the cutoff value. Abbreviations: HR, hazard ratio; CI, confidence interval; ICU, intensive care unit. *Adjusted for CCI, CRP, NRS total score, sex and intervention.*

Supplemental Table S6: Prognostic value of high vs. low levels of glutamine or glutamate on secondary clinical outcomes

	mean (SD)	mean (SD)	unadjusted	adjusted*
**Secondary nutritional outcomes**	**high**	**low**	Coefficient (95% CI) p-value	Coefficient (95% CI) p-value
**Mean caloric intake per day, kcal/kg/d[a]**				
Glutamine	13.59 (10.74)	15.13 (11.14)	91.51 (-110.24 to 293.27) p= 0.372	89.91 (-108.64 to 288.64) p= 0.374
Glutamate	13.68 (11.34)	15.08 (10.86)	38.74 (-163.32 to 240.80) p= 0.706	49.53 (-148.87 to 247.93) p= 0.623
**Mean protein intake per day, kcal/kg/d[a]**				
Glutamine	0.57 (0.43)	0.63 (0.40)	3.81 (-4.05 to 11.67) p= 0.340	4.11 (-3.68 to 11.89) p= 0.299
Glutamate	0.59 (0.40)	0.61 (0.41)	-1.36 (-9.40 to 6.69) p= 0.739	-1.22 (-9.10 to 6.67) p=0.761
**Secondary nutritional outcomes**				
**Reaching caloric-intake goals [b]**				
Glutamine	41/73 (56%)	89/150 (59%)	1.14 (0.65 to 2.00) p= 0.653	1.05 (0.58 to 1.89) p= 0.879
Glutamate	45/72 (63%)	85/151 (56%)	0.77 (0.43 to 1.37) p= 0.380	0.83 (0.46 to 1.51) p= 0.537
**Reaching protein-intake goals [b]**				
Glutamine	30/72 (42%)	75/146 (51%)	1.48 (0.84 to 2.61) p= 0.178	1.52 (0.84 to 2.75) p= 0.163
Glutamate	29/68 (43%)	76/150 (51%)	1.38 (0.78 to 2.46) p= 0.273	1.39 (0.77 to 2.52) p= 0.277

*Cox regression models reporting adjusted hazard ratios according to levels stratified by cutoff values of glutamine (595.5 µmol/l) and glutamate (167.5 µmol/l). Low levels are defined as less or equal the cutoff value, high levels as greater the cutoff value. Abbreviations: HR, hazard ratio; CI, confidence interval. *Adjusted for CCI, CRP, NRS total score, sex and intervention.*

a = per kg of bodyweight until day 10 of hospitalisation

b = refer to achieve 75% of calculated targets

Supplemental Table S7: Prognostic value of high vs. low glutamate-to-glutamine-ratio on mortality

			unadjusted	adjusted *
**Short- and long-term mortality**	n. of event (%)	n. of event (%)	HR (95% CI) p-value	HR (95% CI) p-value
**30-day all-cause mortality**	**high**	**low**		
	17/85 (20%)	40/149 (27%)	1.37 (0.78 to 2.42) p= 0.278	1.25 (0.70 to 2.22) p= 0.448
**180-day all-cause mortality**	**high**	**low**		
	33/85 (39%)	67/149 (45%)	1.24 (0.82 to 1.89) p= 0.308	1.19 (0.78 to 1.81) p= 0.422
**1-year all-cause mortality**	**high**	**low**		
	37/85 (44%)	77/149 (52%)	1.27 (0.86 to 1.88) p= 0.230	1.20 (0.81 to 1.79) p= 0.355
**2-year all-cause mortality**	**high**	**low**		
	42/85 (49%)	94/149 (63%)	1.40 (0.97 to 2.01) p= 0.070	1.34 (0.93 to 1.93) p= 0.115
**3-year all-cause mortality**	**high**	**low**		
	47/85 (55%)	93/149 (62%)	1.26 (0.88 to 1.78) p= 0.204	1.21 (0.85 to 1.73) p= 0.282
**5-year all-cause mortality**	**high**	**low**		
	54/85 (64%)	99/149 (66%)	1.22 (0.87 to 1.70) p= 0.248	1.19 (0.85 to 1.66) p= 0.315

*Cox regression models reporting adjusted hazard ratios according to levels stratified by empirical cutoff value of glutamine-to-glutamate ratio, which is 0.28. Low levels are defined as less or equal the cutoff value, high levels as greater the cutoff value. Abbreviations: HR, hazard ratio; CI, confidence interval. *Adjusted for CCI, CRP, NRS total score, sex and intervention.*

Supplemental Table S8: Prognostic value of high vs. low glutamate-to-glutamine-ratio on secondary clinical outcomes

			unadjusted	adjusted
**Secondary clinical outcomes**	n. of event (%)	n. of event (%)	OR (95% CI) p-value	OR (95% CI) p-value
**Adverse events, 30 days**	**high**	**low**		
	28/85 (33%)	63/149 (42%)	1.49 (0.85 to 2.60) p= 0.160	1.34 (0.75 to 2.42) p= 0.3234
**ICU, 30 days**	**high**	**low**		
	0/85 (0%)	4/149 (3%)	1.00	1.00
**Rehospitalisation 30 days**	**high**	**low**		
	3/85 (4%)	15/149 (10%)	3.06 (0.86 to10.89) p= 0.084	2.99 (0.83 to 10.71) p= 0.093
**Major complications**	**high**	**low**		
	5/85 (6%)	17/149 (11%)	2.06 (0.73 to 5.80) p= 0.171	2.06 (0.72 to 5.84) p= 0.176
**Decline in functional status of >10%**	**high**	**low**		
	20/85 (24%)	44/149 (30%)	1.36 (0.74 to 2.51) p= 0.323	1.19 (0.61 to 2.30) p= 0.616
**Falls, 180 days**	**high**	**low**		
	6/85 (7%)	17/148 (11%)	1.71 (0.65 to 4.51) p= 0.280	1.89 (0.70 to 5.10) p= 0.209

*Cox regression models reporting adjusted hazard ratios according to levels stratified by empirical cutoff value of glutamine-to-glutamate ratio, which is 0.28. Low levels are defined as less or equal the cutoff value, high levels as greater the cutoff value. Abbreviations: OR, odds ratio; CI, confidence interval; ICU, intensive care unit. *Adjusted for CCI, CRP, NRS total score, sex and intervention.*

Supplemental Table S9: Prognostic value of high vs. low glutamate-to-glutamine-ratio on secondary clinical outcomes

			unadjusted	adjusted
**Secondary nutritional outcomes**	mean (SD)	mean (SD)	Coefficient (95% CI) p-value	Coefficient (95% CI) p-value
**Mean caloric intake per day, kcal/kg/d [a]**	**high**	**low**		
	14.28 (11.33)	14.83 (10.86)	-23.72 (-220.10 to 172.65) p= 0.812	-6.42 (-199.55 to 186.72) p= 0.948
**Mean protein intake per day, kcal/kg/d [a]**	**high**	**low**		
	0.62 (0.40)	0.60 (0.42)	-3.87 (-11.61 to 3.88) p= 0.326	-3.44 (-11.04 to 4.16) p= 0.373
**Secondary nutritional outcomes**	n. of event (%)	n. of event (%)	OR (95% CI) p-value	OR (95% CI) p-value
**Reaching caloric-intake goals [b]**	**high**	**low**		
	48/82 (59%)	82/141 (58%)	0.98 (0.57 to 1.71) p= 0.956	1.05 (0.59 to 1.87) p= 0.864
**Reaching protein-intake goals [b]**	**high**	**low**		
	33/78 (42%)	72/140 (51%)	1.44 (0.83 to 2.52) p= 0.197	1.47 (0.83 to 2.62 p= 0.188

*Cox regression models reporting adjusted hazard ratios according to levels stratified by empirical cutoff value of glutamine-to-glutamate ratio, which is 0.28. Low levels are defined as less or equal the cutoff value, high levels as greater the cutoff value. Abbreviations: OR, odds ratio; CI, confidence interval. *Adjusted for CCI, CRP, NRS total score, sex and intervention.*
